# Supplementary material for: Gastric Inflammation Impacts Serotonin Secretion in a Mouse Model of Helicobacter pylori Vaccination
Source: Int J Mol Sci. 2025 Aug 10;26(16):7735. doi: 10.3390/ijms26167735 (PMC12386332; doi:10.3390/ijms26167735)
Supplement: Supplementary file 1 [file ijms-26-07735-s001.zip › ijms-3654276-supplementary.pdf]

Supplementary Figures.

Supplementary Figure S1.

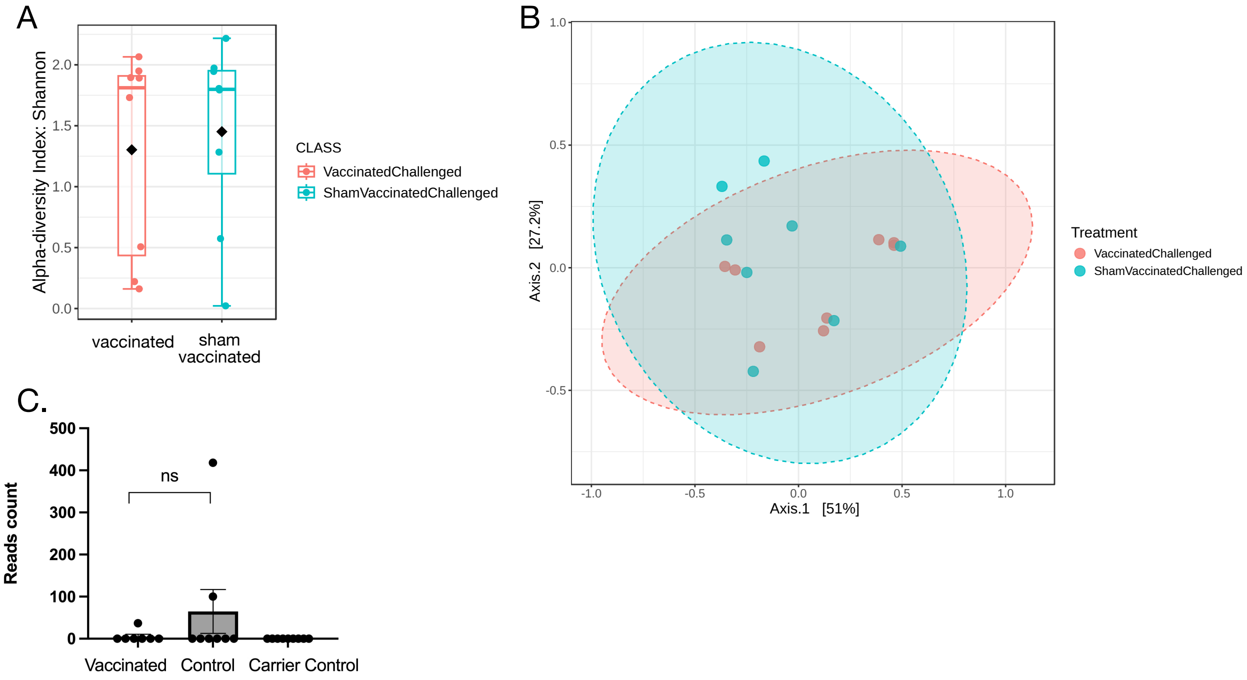

Supplementary Figure S1. Effect of *H. pylori* infection and vaccination on gastric microbiota. DNA was extracted from gastric contents of, vaccinated and sham vaccinated *H. pylori* challenged mice at day 21 post challenge, and processed for 16S RNA gene sequencing. A. *H. pylori* infection or vaccination did not cause an overall significant change in alpha diversity (genus level, Shannon diversity Index). B. Overall beta diversity was also not significantly changed. PCoA, Bray Curtis Index, explaining 51 and 27.2% of variation in the samples). C. Inspection of the read data revealed that *Helicobacter* spp. were detected at low counts, mainly in the sham vaccinated group, but these reads were at low abundance and were filtered out of further analysis.

Supplementary Figure S2.

Figure S2. Primer sequences using in this study

**Gene of interest**	**Species**	**Forward Primer (5′---3′)**	**Reverse primer (5′-3′)**
IL-8	Human	AGGCCAAGGGCCAAGAGAAT	AGGACTTGTGGATCCTGGCT
HTR1A	Mouse	TGCGCTGATCTCGCTCACTT	CGCCCATAGAGGACCAGCAT
HTR1A	Human	TGCCCTTCTTCATCGTGGCT	TCATCACTGGCGGCAGAACT
HTR1B	Mouse	GCACTAAGTCTGTGGCAGCG	TCTCTCAGGTTCCCTTGTCCCT
HTR1B	Human	CTGCTGGTTCCACCTAGCCA	TTGGTCCCCAAAGGTCGCTT
HTR2B	Mouse	CCCTGTGTCCTGCCTGGTTAT	CTGGGATGGCGATGCCTATTG
HTR2B	Human	TGGCATGCAAGTTCAAACACG	GAGCGCATACACACATCTGTCC
β-actin (reference gene)	Human	AGAAAATCTGGCACCACACC	AACGGCAGAAGAGACAACCA
HPRT (reference gene)	Mouse	GTTGGATACAGGCCAGCATTTGT	CACAGGACTAGAACACCTGC
